# Relationships among Socioeconomic Factors and Self-rated Health in Japanese Adults: NIPPON DATA2010

**DOI:** 10.2188/jea.JE20170246

**Published:** 2018-03-05

**Authors:** Atsuhiko Ota, Hiroshi Yatsuya, Nobuo Nishi, Nagako Okuda, Takayoshi Ohkubo, Takehito Hayakawa, Aya Kadota, Akira Okayama, Katsuyuki Miura

**Affiliations:** 1 Department of Public Health, Fujita Health University School of Medicine, Aichi, Japan; 2 International Center for Nutrition and Information, National Institute of Health and Nutrition, National Institutes of Biomedical Innovation, Health and Nutrition, Tokyo, Japan; 3 Department of Health and Nutrition, University of Human Arts and Sciences, Saitama, Japan; 4 Department of Hygiene and Public Health, Teikyo University School of Medicine, Tokyo, Japan; 5 Research Center for Social Studies of Health and Community, Ritsumeikan University, Kyoto, Japan; 6 Center for Epidemiologic Research in Asia, Shiga University of Medical Science, Shiga, Japan; 7 Department of Public Health, Shiga University of Medical Science, Shiga, Japan; 8 Research Institute of Strategy for Prevention, Tokyo, Japan

**Keywords:** socioeconomic status (SES), self-rated health (SRH), Japan, NIPPON DATA2010, cross-sectional study

## Abstract

**Background:**

The distributions of socioeconomic status (SES) factors have been changing in Japan. We examined the relationships among SES and self-rated health (SRH) in Japanese adults.

**Methods:**

We analyzed 1,178 men and 1,555 women. We showed the distribution of SRH by sex and age and examined cross-sectional relationships among educational attainment, marital/living statuses, working status, household income and expenditure, and fine SRH (defined as excellent, very good, or good). We adjusted for age, subjective symptoms, visiting doctors, monthly equivalent household expenditure (EHE), and living in their own house.

**Results:**

The age-standardized prevalence of fine SRH was 79% and 73% among men and women, respectively. Among men, graduating from high school (adjusted odds ratio [aOR] 1.53; 95% confidence interval [CI], 1.07–2.19, relative to graduating from elementary or junior high school) and university or junior college (aOR 1.74; 95% CI, 1.15–2.62) was associated with fine SRH. Among women, graduating from university or junior college was associated with fine SRH (aOR 1.65; 95% CI, 1.12–2.46). Neither marital/living status nor working status was associated with SRH after adjustments for age in either sex. Among women, high EHE and income were associated with fine SRH (the highest expenditure group: aOR 1.80; 95% CI, 1.22–2.65; the highest income group: aOR 2.15; 95% CI, 1.34–3.46, relative to the corresponding lowest group). These simple relationships were not observed for men.

**Conclusions:**

High educational attainment was associated with fine SRH. Relationships among household income, EHE, and fine SRH differed by sex.

## INTRODUCTION

Self-rated health (SRH), a subjective perception of an individual’s overall health, is a powerful predictor of all-cause mortality in general populations.^1^^,^^2^ SRH is typically assessed using a single question that asks the respondents to rate their overall health on a scale from excellent to poor.^3^ The SRH of the general population in Japan has been officially assessed every 3 years in the Comprehensive Survey of Living Conditions (CSLC), a nationwide cross-sectional survey conducted in a representative general population. According to the CSLC conducted in June 2010 (CSLC2010), the prevalence of Japanese adults with fine SRH defined as excellent, very good, or good SRH was 74% and 71% among men and women, respectively.^4^

Socioeconomic statuses (SESs) are reported as fundamental determinants of human function across the life span.^5^ Lower educational attainment,^6^^,^^7^ being unmarried,^7^^,^^8^ not working,^9^^,^^10^ and having a low income^6^^,^^10^^–^^13^ have been associated with a deteriorated SRH in Japan; however, previous findings are inconsistent.^9^^,^^14^^,^^15^

The distributions of SESs have been changing in Japan for dozens of years. More individuals have attained a high-level education.^16^ The numbers of women and elderly people in the labor force have been growing.^17^^,^^18^ Mean annual household income has been decreasing.^19^ Marriage rates have also been declining.^20^ Couples are getting married later and later.^20^ The average number of members per household has been in a steady decline.^21^ These changes in SESs may be followed by changes in the class identification, a subjective view of their location in the social structure and their social preferences.^22^ Since low-grade class identification was reportedly associated with deteriorated SRH in Japan,^14^ further studies are needed in order to clarify whether SESs are associated with SRH in Japan.

In the present study, we examined relationships among educational attainment, marital/living status, working status, household income and expenditure, and SRH using the latest cross-sectional dataset of a representative Japanese general population.

## METHODS

### Study population

A prospective cohort study on cardiovascular diseases entitled the National Integrated Project for Prospective Observation of Non-communicable Disease and its Trends in the Aged 2010 (NIPPON DATA2010), was launched in November 2010, collaborating with the National Health and Nutrition Survey in November 2010 (NHNS2010) and CSLC2010, which were executed by the Ministry of Health, Labour, and Welfare, Japan. The methodology and summary of the findings of the NIPPON DATA2010, NHNS2010, and CSLC2010 have been reported elsewhere in more detail.^23^^–^^26^

The participants in the dietary survey for NHNS2010 were 8,815 residents who were aged 1 year and older and from 300 districts randomly selected throughout Japan. Among 7,229 participants aged 20 years or older, 2,898 (1,239 men and 1,659 women) agreed to participate in the baseline survey for NIPPON DATA2010. In the present study, we integrated the data of NIPPON DATA2010 with those of NHNS2010 and CSLC2010 for 2,807 participants (1,207 men and 1,600 women). Based on the information obtained from CSLC, we excluded 74 participants who had been admitted to a hospital or living in a facility at the time of the CSLC2010 survey. We eventually analyzed the data of the remaining 2,733 subjects (1,178 men and 1,555 women).

All participants gave their informed consent before enrollment. The study plan was approved by the Institutional Review Board of Shiga University of Medical Science (No. 22-29, 2010).

### SRH

Data on SRH was obtained through a question item, “What is your present general health status?” in the self-reported questionnaire for CSLC2010. The following five options were given: “excellent,” “very good,” “good,” “fair,” and “poor”. Subjects who did not choose any of these options were regarded as returning an invalid response. Subjects were dichotomized according to their responses; those who self-reported “excellent”, “very good”, or “good” were regarded as being in a fine SRH condition, while those who reported “fair” or “poor” or gave an invalid response were regarded as being in a deteriorated SRH condition.

### SES

In the present study, SES variables included educational attainment (graduating from elementary or junior high school, high school, or university or junior college), marital/living status (married, single and not living alone, or single and living alone), yearly household income (less than two, two through six, or greater than six million Japanese Yen (JPY), or returning an invalid response; 100 JPY is nearly equivalent to 0.9 US Dollars as of the beginning of May 2017), and monthly equivalent household expenditure (EHE; calculated as monthly household expenditure divided by the square root of the number of household members) in May 2010 (in quintiles). The above classification was made based on information obtained using self-administered questionnaires for NIPPON DATA2010 (educational attainment), CSLC2010 (marital status, the number of household members, occupation, and monthly EHE), and NHNS2010 (household income).

### Possible confounders in the relationship between SES and SRH

Information on whether subjects had any subjective symptoms and were visiting a doctor was obtained through a self-administered questionnaire for CSLC2010. Subjects were asked whether they had any of the following symptoms: fever, tiredness, not sleeping well, feeling irritated, forgetfulness, headaches, dizziness, blurred vision, vision problems, ear buzzing, hearing difficulty, palpitation, breath shortness, chest pain, cough and/or sputum, congested and/or running nose, wheezing, heavy stomach and/or heartburn, diarrhea, constipation, appetite loss, abdominal and/or stomach pain, pain and/or bleeding due to hemorrhoids, tooth pain, swelling of and/or bleeding from dental gums, difficulty biting, rash, itchiness, shoulder stiffness, low back pain, pain at limb joints, difficulty in limb movement, feeling cold at the limbs, swelling and/or tiredness at the foot, difficulty and/or pain in urination, urinary incontinence, menses issues, bone fracture and/or joint injury, cut and/or burn injury, and other symptoms, including those yet to be diagnosed. In addition, subjects were asked whether they were visiting a doctor for any of the following health issues: diabetes; obesity; dyslipidemia; thyroid diseases; depression and/or other mental disorders; Parkinson disease; neurological diseases; vision disorders; hearing disorders; hypertension; stroke; coronary artery diseases; other circulatory diseases; cold; allergic rhinitis; asthma; other respiratory diseases; diseases of the stomach, duodenum, liver, gall bladder, or other digestive organs; dental diseases; atopic dermatitis; other skin disorders; gout; rheumatoid arthritis; arthropathy; shoulder stiffness; low back pain; osteoporosis; renal diseases; prostatic hyperplasia; menopause and post-menopause disorders; bone fracture; other injuries; anemia and/or hematological diseases; cancer; pregnancy and/or obstetric disorders; infertility; and other health issues, including those yet to be diagnosed.

### Statistical analysis

The distribution of SRH conditions was assessed separately by sex and according to 10-year age groups. The Cochran-Armitage test was employed to test linear associations between age and fine SRH. The proportions of fine SRH were age-standardized to the national population of Japan in 2010 (National Population Census).^27^

We calculated odds ratios (ORs) and their 95% confidence intervals (CIs) for fine SRH by each SES variable with logistic regression analyses. We initially calculated crude ORs. When examining the relationship between yearly household income and SRH, we adjusted for the square root-transformed number of household members. When examining the relationship between monthly EHE and SRH, we adjusted for whether or not subjects were living in their own house because EHE included household rent but not a mortgage. After calculating age-adjusted ORs, we constructed two multivariable models, models 1 and 2. In model 1, we adjusted for age, having any subjective symptoms, and visiting a doctor. In model 2, we adjusted for monthly EHE and living in their own house, in addition to the factors in model 1. In order to assist in the interpretation of relationships among each SES variable and fine SRH, we also examined the relationships among age and each SES variable using the chi-squared test separately by sex.

Statistical calculations were performed using IBM SPSS Statistics 24 for Windows (IBM Japan, Tokyo, Japan). The level of significance was 0.05 (two-tailed) for all tests. Subjects with missing responses were excluded from the corresponding analyses.

## RESULTS

### Prevalence of SRH

The prevalence of each SRH category is presented separately by sex and age in Table 1. The older the subjects’ age group, the lower the prevalence of fine SRH in men and women (*P* for trend <0.001). The age-standardized prevalence of fine SRH was 79% in men and 73% in women.

**Table 1.** Self-rated health conditions of subjects: NIPPON DATA2010

Age, years	*N*	Point prevalence of SRH						

		Fine (excellent, very good, or good)	Excellent	Very good	Good	Fair	Poor	Invalid response
Men								
20–29	51	92%	33%	12%	47%	6%	0%	2%
30–39	106	88%	25%	21%	42%	8%	1%	4%
40–49	122	89%	28%	16%	45%	9%	0%	2%
50–59	189	79%	14%	15%	50%	10%	2%	9%
60–69	354	69%	12%	15%	42%	12%	1%	18%
70–79	264	60%	10%	8%	42%	14%	2%	23%
≥80	92	59%	10%	11%	38%	13%	2%	26%
Total	1,178	72%	15%	14%	44%	11%	1%	15%

Women								
20–29	62	77%	24%	15%	39%	13%	3%	6%
30–39	224	85%	23%	24%	39%	8%	2%	5%
40–49	179	81%	15%	21%	45%	12%	1%	7%
50–59	267	79%	12%	17%	50%	10%	1%	10%
60–69	407	65%	11%	11%	43%	13%	2%	21%
70–79	315	56%	7%	11%	38%	14%	3%	26%
≥80	101	58%	3%	14%	42%	15%	2%	25%
Total	1,555	70%	12%	15%	43%	12%	2%	16%

SRH, self-rated health.

Age-standardized point prevalence of a fine SRH condition: 79.1% (men) and 72.7% (women). The 2010 Japan National Census Population was used as a reference.

*P* for trend <0.001 was found for the relationship between age and the point prevalence of fine SRH in men and women (the Cochran-Armitage test for trends).

### Relationships among SES and SRH in men

Educational attainment was positively associated with SRH (Table 2). A bivariate analysis (dependent variable being fine SRH or not) showed a relationship between marital/living status and fine SRH. The relationship between these two factors was inconsistent after adjustment for age, having any subjective symptoms, visiting a doctor, monthly EHE, and living in their own house. Working status was significantly associated with fine SRH; however, the relationship did not remain significant after adjustment for age. Yearly household income was also positively associated with fine SRH. The association between an invalid response and fine SRH was insignificant after adjustment for age. The positive association between a 2–6 million JPY household income and fine SRH relative to less than 2 million JPY remained significant even after adjustments for age, having any subjective symptoms, visiting a doctor, monthly EHE, and living in their own house. Regarding monthly EHE, only those at the 4^th^ highest quintile of EHE showed an association with fine SRH relative to those at the lowest quintile.

**Table 2.** Relationships among socioeconomic status and fine self-rated health in men (*n* = 1,178): NIPPON DATA2010

SES	*N*	Prevalence of fine SRH	Crude OR (95% CI)	Age-adjusted OR (95% CI)	Model 1	Model 2
	
					Adjusted OR (95% CI)	Adjusted OR (95% CI)
Educational attainment^i^						
Elementary or junior high school	290	169 (58%)	1	1	1	1
High school	496	374 (75%)	2.19 (1.61–2.99)^***^	1.58 (1.13–2.19)^**^	1.55 (1.10–2.20)^*^	1.53 (1.07–2.19)^*^
University or junior college	383	306 (80%)	2.85 (2.02–4.01)^***^	1.74 (1.20–2.51)^**^	1.76 (1.19–2.59)^**^	1.74 (1.15–2.62)^**^

Marital/living status						
Married	953	687 (72%)	1	1	1	1
Single, not living alone	112	93 (83%)	1.90 (1.13–3.17)^*^	1.07 (0.60–1.89)	1.01 (0.55–1.83)	0.93 (0.51–1.70)
Single, living alone	113	74 (65%)	0.73 (0.49–1.11)	0.66 (0.43–1.01)	0.63 (0.40–0.99)^*^	0.65 (0.40–1.07)

Working status						
Working	718	568 (79%)	1	1	1	1
Not working, including housemaking exclusively	460	286 (62%)	0.43 (0.33–0.56)^***^	0.75 (0.55–1.02)	0.75 (0.54–1.05)	0.72 (0.51–1.01)

Yearly household income, million JPY^a^						
Less than 2	195	113 (58%)	1	1	1	1
2 through 6	655	488 (75%)	2.05 (1.46–2.88)^***^	1.90 (1.34–2.70)^***^	1.82 (1.26–2.63)^**^	1.64 (1.10–2.44)^*^
Greater than 6	224	178 (79%)	2.63 (1.67–4.12)^***^	1.99 (1.24–3.18)^**^	1.84 (1.13–3.02)^*^	1.59 (0.94–2.70)
Invalid response	104	75 (72%)	1.76 (1.03–2.98)^*^	1.56 (0.90–2.71)	1.50 (0.84–2.68)	1.38 (0.76–2.52)

Monthly equivalent household expenditure^b,ii^						
1st quintile (Lowest)	217	148 (68%)	1	1	1	
2nd quintile	244	175 (72%)	1.18 (0.79–1.76)	1.18 (0.78–1.80)	1.15 (0.74–1.79)	
3rd quintile	238	170 (71%)	1.17 (0.78–1.74)	1.22 (0.80–1.86)	1.18 (0.76–1.83)	
4th quintile	216	172 (80%)	1.82 (1.18–2.82)^**^	2.08 (1.32–3.28)^**^	1.91 (1.19–3.08)^**^	
5th quintile (Highest)	214	156 (73%)	1.26 (0.83–1.90)	1.32 (0.86–2.03)	1.32 (0.84–2.07)	

CI, confidence interval; OR, odds ratio; SES, socioeconomic status; SRH, self-rated health.

*P* value: ^*^<0.05, ^**^<0.01, ^***^<0.001.

ORs were calculated using multiple logistic regression analyses, adjusted for age, having any subjective symptoms, and visiting a doctor in Model 1 and additionally adjusted for monthly equivalent household expenditure and living in their own house in Model 2.

^a^The square root-transformed number of household members was adjusted.

^b^Living in their own house was adjusted.

Number of missing responses: i = 9; ii = 49.

### Relationships among SES and SRH in women

The relationship between having a high school diploma and fine SRH was significantly attenuated after adjustment for age (Table 3). However, the relationship between having a university or junior college diploma and fine SRH remained significant even after adjustments for age, any subjective symptoms, visiting a doctor, monthly EHE, and living in their own house. The inverse association between being single and living alone and fine SRH was not independent of age, having any subjective symptoms, visiting a doctor, monthly EHE, and living in their own house. Although working women were more likely to have fine SRH, this relationship was attenuated after age adjustments. In contrast to men, women in the lowest yearly household income category were the least likely to have fine SRH relative to other income categories, including that with an invalid response, independent of confounding variables. Furthermore, high (the 4^th^ and 5^th^ highest quintile) monthly EHE was significantly associated with fine SRH.

**Table 3.** Relationships among socioeconomic status and fine self-rated health in women (*n* = 1,555): NIPPON DATA2010

SES	*N*	Prevalence of fine SRH	Crude OR (95% CI)	Age-adjusted OR (95% CI)	Model 1	Model 2
	
					Adjusted OR (95% CI)	Adjusted OR (95% CI)
Educational attainment^i^						
Elementary or junior high school	366	213 (58%)	1	1	1	1
High school	708	488 (69%)	1.59 (1.23–2.07)^***^	1.23 (0.94–1.62)	1.14 (0.86–1.53)	1.02 (0.75–1.38)
University or junior college	477	393 (82%)	3.36 (2.45–4.60)^***^	1.95 (1.37–2.79)^***^	1.87 (1.29–2.70)^***^	1.65 (1.12–2.46)^*^

Marital/living status						
Married	1,100	803 (73%)	1	1	1	1
Single, not living alone	258	178 (69%)	0.82 (0.61–1.11)	0.86 (0.62–1.19)	0.84 (0.60–1.18)	0.84 (0.59–1.20)
Single, living alone	197	114 (58%)	0.51 (0.37–0.69)^***^	0.75 (0.53–1.05)	0.70 (0.49–1.00)^*^	0.77 (0.53–1.13)

Working status						
Working	672	522 (78%)	1	1	1	1
Not working, including housemaking exclusively	883	573 (65%)	0.53 (0.42–0.67)^***^	0.80 (0.62–1.04)	0.81 (0.62–1.07)	0.76 (0.57–1.01)

Yearly household income, million JPY^a^						
Less than 2	296	165 (56%)	1	1	1	1
2 through 6	794	568 (72%)	1.85 (1.39–2.47)^***^	1.66 (1.24–2.23)^***^	1.70 (1.25–2.32)^***^	1.53 (1.09–2.14)^*^
Greater than 6	294	240 (82%)	3.13 (2.10–4.64)^***^	2.34 (1.55–3.54)^***^	2.39 (1.55–3.69)^***^	2.15 (1.34–3.46)^**^
Invalid response	171	122 (71%)	1.78 (1.17–2.71)^**^	1.71 (1.12–2.63)^*^	1.94 (1.24–3.04)^**^	2.19 (1.36–3.53)^**^

Equivalent monthly household expenditure^b,ii^						
1st quintile (Lowest)	278	175 (63%)	1	1	1	
2nd quintile	281	197 (70%)	1.39 (0.97–1.98)	1.26 (0.88–1.82)	1.40 (0.95–2.06)	
3rd quintile	320	226 (71%)	1.42 (1.01–2.00)^*^	1.25 (0.87–1.79)	1.30 (0.89–1.90)	
4th quintile	289	217 (75%)	1.78 (1.24–2.55)^**^	1.57 (1.08–2.29)^*^	1.74 (1.18–2.59)^**^	
5th quintile (Highest)	304	224 (74%)	1.64 (1.16–2.34)^**^	1.48 (1.03–2.14)^*^	1.80 (1.22–2.65)^**^	

CI, confidence interval; OR, odds ratio; SES, socioeconomic status; SRH, self-rated health.

*P* value: ^*^<0.05, ^**^<0.01, ^***^<0.001.

ORs were calculated using multiple logistic regression analyses, adjusted for age, having any subjective symptoms, and visiting a doctor in Model 1 and additionally adjusted for monthly equivalent household expenditure and living in their own house in Model 2.

^a^The square root-transformed number of household members was adjusted.

^b^Living in their own house was adjusted.

Number of missing responses: i = 4; ii = 83.

### Relationships among age, sex, and SES

Significant differences were observed in educational attainment, marital/living status, working status, and yearly household income by age in men and women (eTable 1 and eTable 2). Age was correlated with monthly EHE in women only.

## DISCUSSION

### Prevalence of fine SRH

The age-standardized prevalence of fine SRH was 79% and 73% among men and women, respectively. The present study indicated that the prevalence of fine SRH decreased as subjects aged. These results are consistent with those among all adult participants in CSLC2010.^4^ Therefore, the subjects in the present study may be regarded as being representative of the participants of CSLC2010.

### Educational attainment and SRH

In the present study, a significant association was observed between a younger age and higher educational attainment. This needs to be considered when interpreting results on the relationship between educational attainment and SRH. Among men, the OR for the relationship between educational attainment of high school or higher and fine SRH was attenuated by approximately 30% after adjustments for age, even though it remained significant. On the other hand, in women, only university or junior college diplomas were significantly associated with fine SRH after adjusting for age.

Previous findings in Japan on the relationship between educational attainment and SRH are inconsistent. Honjo et al also reported that, relative to an 11-year or shorter educational duration, a 13-year or longer educational duration was associated with a lower prevalence of poor self-rated physical health in women only, after adjustments for age, gender, and marital status.^6^ Meanwhile, Wada et al examined male employees in their fifties and found that lower educational attainment was associated with worse SRH, independent of work-related conditions and marital status.^7^ In contrast, some research groups failed to find a significant association between educational attainment and SRH.^9^^,^^14^ The strength of the present study is that we adjusted for any subjective symptoms and visiting a doctor in the assessment of the relationship between educational attainment and SRH, which had not been performed in the above-described studies.

### Marital/living status and SRH

Men who were single and were not living alone appeared to have better SRH than those who were married, while women who were single and were living alone appeared to have worse SRH. These relationships diminished after age adjustments. In the present study, most men who were single and were not living alone were young, ranging from 20–49 years old. Most women who were single and were living alone were aged 60 or older. Age is considered to be a major confounding factor in the relationship between marital/living status and SRH.

Further studies are needed in order to clarify whether marital/living status is related to SRH independent of age and other confounding factors. In a study by Wada et al, in which subjects were Japanese male employees aged between 50 and 59 years, married men had better SRH than unmarried men independent of educational attainment and the types of occupations and employment contracts.^7^ We did not obtain information on the human relationships of the subjects with their spouse and family, which may be a health determinant. Chung and Kim reported that satisfaction in marriage was associated with fine SRH in Japan, independent of sex, age, educational attainment, employment, self-rated social class, and religion.^15^

### Working status and SRH

Men and women who were working appeared to self-rate their health statuses as better than those who were not. However, these relationships vanished following age adjustments. The younger the subjects were in the present study, the more frequently they were working. Therefore, this relationship may merely be a result of an age confounder.

On the other hand, previous studies implied that different findings may have been observed if another work-related variable, such as occupation contents or work contracts, had been used to classify subjects. For example, Furuya et al reported that the employed were more likely to have good SRH than the unemployed among community dwellers, even after adjustments for gender, age, educational attainment, and household income.^9^ Wada et al showed that middle-aged male employees in manufacturing were more likely to experience the worsening of their SRH, independent of educational attainment and marital status, than those in management.^7^ They also reported that precarious employment contracts were associated with worsening SRH.^7^ Further studies are needed in order to clarify the relationship between work-related variables and SRH.

### Household income, EHE, and SRH

The relationships among yearly household income, monthly EHE, and SRH differed by sex. In women, a high income and EHE were consistently associated with fine SRH. Meanwhile, in men, the highest income group was not associated with fine SRH after adjustment for EHE. Furthermore, the highest expenditure group was not associated with fine SRH. Other studies also reported the possible effect modification by sex for the relationships among income, EHE, and health in Japan. In a study by Honjo et al, a relationship between a low income and poor self-rated physical and mental health was only found in women.^6^ Fukuda and Hiyoshi reported that, in women, lower EHE was associated with cardiovascular risk factors, such as obesity, hypertension, diabetes, and the presence of multiple risk factors, while this relationship was not found in men.^28^ Men generally earn more than women in Japan. The mean monthly paid income of husbands was 422,000 JPY, while that of wives was 136,000 JPY, according to the Family Income and Expenditure Survey of Japan in 2010.^29^ Thus, husbands likely earned high incomes in high-income households, and the SRH of these husbands may have worsened due to the high burden of work.

A considerable number of subjects gave invalid responses regarding their household income (10.1% of all subjects) and EHE (4.8%). Concerning the household income, we made an independent group of those with an invalid response and included it in statistical analyses. Among women, this group showed a higher prevalence of fine SRH than the lowest income group. In contrast, this relationship was not found in men after adjustments for age. Difficulties may be associated with calculating the household income in a household in which two or more individuals earn an income. Concurrently, some of these households must have earned incomes of 2 million JPY or greater. This may explain the present results showing that women who gave invalid responses regarding their household income reported fine SRH more frequently than those with yearly household incomes of less than 2 million JPY.

### Limitations

The present study has the following limitations. We were unable to clarify the causal relationship between SES and SRH because the present results were obtained in a cross-sectional manner. Furthermore, there was a possible selection bias. The present subjects were limited to those who were willing to participate in the study and were not staying in a hospital or living in a care facility. The present results may not be applicable to the general population. Therefore, these results may not be applicable to other countries in which SES and SRH are different from Japan.

### Conclusion

The age-standardized prevalence of fine SRH was 79% and 73% among men and women, respectively. The older the subjects’ age category, the lower the prevalence of fine SRH. We analyzed relationships among SES and SRH using the latest cross-sectional dataset of a representative Japanese general population. Higher educational attainment was positively associated with SRH. In men, graduating from high school or higher was associated with fine SRH relative to graduating from elementary or junior high school, while, in women, graduating from university or junior college, but not from high school, was associated with fine SRH. The relationships among marital/living status, working status, and SRH were explained by differences in age in the present study. In addition, relationships among yearly household income, monthly EHE, and SRH differed by sex. The highest income and EHE groups were associated with fine SRH in women. However, these simple relationships were not found in men.

## APPENDIX A. SUPPLEMENTARY DATA

eTable 1. Relationships among age and socioeconomic status in men (n = 1,178): NIPPON DATA2010

eTable 2. Relationships among age and socioeconomic status in women (n = 1,555): NIPPON DATA2010
