# Supplementary material for: Relationships among Socioeconomic Factors and Self-rated Health in Japanese Adults: NIPPON DATA2010
Source: J Epidemiol. 2018 Mar 5;28(Suppl 3):S66–72. doi: 10.2188/jea.JE20170246 (PMC5825690; doi:10.2188/jea.JE20170246)
Supplement: Supplementary file 1 [file je-28-S066-s001.pdf]

eTable 1. Relationships among age and socioeconomic status in men (n=1,178): NIPPON DATA2010

SES	Age, years N	20–29 51	30–39 106	40–49 122	50–59 189	60–69 354	70–79 264	≥80 92	P value
Educational attainment[i]									
Elementary or junior high school		3 (6%)	3 (3%)	3 (2%)	25 (13%)	99 (28%)	103 (39%)	54 (59%)	<0.001
High school		18 (35%)	46 (43%)	58 (48%)	87 (46%)	169 (48%)	102 (39%)	16 (17%)	
University or junior college		30 (59%)	56 (53%)	61 (50%)	75 (40%)	83 (23%)	57 (22%)	21 (23%)	
Marital/living status									
Married		11 (22%)	79 (75%)	94 (77%)	159 (84%)	297 (84%)	236 (89%)	77 (84%)	<0.001
Single, not living alone		34 (67%)	17 (16%)	18 (15%)	11 (6%)	16 (5%)	7 (3%)	9 (10%)	
Single, living alone		6 (12%)	10 (9%)	10 (8%)	19 (10%)	41 (12%)	21 (8%)	6 (7%)	
Working status									
Working		43 (84%)	102 (96%)	116 (95%)	170 (90%)	197 (56%)	78 (30%)	12 (13%)	<0.001
Not working, including housemaking exclusively		8 (16%)	4 (4%)	6 (5%)	19 (10%)	157 (44%)	186 (70%)	80 (87%)	
Yearly household income, million JPY									
Less than 2		2 (4%)	6 (6%)	10 (8%)	27 (14%)	76 (21%)	57 (22%)	17 (18%)	<0.001
2 through 6		28 (55%)	70 (66%)	56 (46%)	90 (48%)	195 (55%)	164 (62%)	52 (57%)	
Greater than 6		10 (20%)	22 (21%)	46 (38%)	60 (32%)	46 (13%)	28 (11%)	12 (13%)	
Invalid response		11 (22%)	8 (8%)	10 (8%)	12 (6%)	37 (10%)	15 (6%)	11 (12%)	
Equivalent monthly household expenditure[ii]									
1st quintile (Lowest)		8 (16%)	20 (19%)	26 (21%)	34 (18%)	71 (20%)	40 (15%)	18 (20%)	0.436
2nd quintile		13 (25%)	33 (31%)	21 (17%)	39 (21%)	66 (19%)	50 (19%)	22 (24%)	
3rd quintile		12 (24%)	23 (22%)	22 (18%)	35 (19%)	74 (21%)	57 (22%)	15 (16%)	
4th quintile		7 (14%)	12 (11%)	21 (17%)	41 (22%)	61 (17%)	62 (23%)	12 (13%)	
5th quintile (Highest)		10 (20%)	16 (15%)	23 (19%)	33 (17%)	66 (19%)	46 (17%)	20 (22%)	

JPY, Japanese yen; SES, socioeconomic status.
Number of missing responses: i = 9; ii = 49.
P values were calculated with the chi-squared test.

eTable 2. Relationships among age and socioeconomic status in women (n=1,555): NIPPON DATA2010

SES	Age, years<br>N	20–29<br>62	30–39<br>224	40–49<br>179	50–59<br>267	60–69<br>407	70–79<br>315	≥80<br>101	P value
Educational attainment[i]									
Elementary or junior high school		7 (11%)	7 (3%)	10 (6%)	25 (9%)	127 (31%)	144 (46%)	46 (46%)	<0.001
High school		23 (37%)	83 (37%)	71 (40%)	130 (49%)	222 (55%)	133 (42%)	46 (46%)	
University or junior college		32 (52%)	134 (60%)	98 (55%)	111 (42%)	58 (14%)	37 (12%)	7 (7%)	
Marital/living status									
Married		14 (23%)	185 (83%)	146 (82%)	225 (84%)	320 (79%)	184 (58%)	26 (26%)	<0.001
Single, not living alone		47 (76%)	31 (14%)	26 (15%)	27 (10%)	46 (11%)	49 (16%)	32 (32%)	
Single, living alone		1 (2%)	8 (4%)	7 (4%)	15 (6%)	41 (10%)	82 (26%)	43 (43%)	
Working status									
Working		45 (73%)	126 (56%)	127 (71%)	184 (69%)	136 (33%)	51 (16%)	3 (3%)	<0.001
Not working, including housemaking exclusively		17 (27%)	98 (44%)	52 (29%)	83 (31%)	271 (67%)	264 (84%)	98 (97%)	
Yearly household income, million JPY									
Less than 2		10 (16%)	18 (8%)	11 (6%)	31 (12%)	88 (22%)	104 (33%)	34 (34%)	<0.001
2 through 6		26 (42%)	129 (58%)	84 (47%)	124 (46%)	232 (57%)	148 (47%)	51 (50%)	
Greater than 6		13 (21%)	57 (25%)	69 (39%)	83 (31%)	40 (10%)	28 (9%)	4 (4%)	
Invalid response		13 (21%)	20 (9%)	15 (8%)	29 (11%)	47 (12%)	35 (11%)	12 (12%)	
Equivalent monthly household expenditure[ii]									
1st quintile (Lowest)		8 (13%)	44 (20%)	16 (9%)	35 (13%)	70 (17%)	87 (28%)	18 (18%)	<0.001
2nd quintile		11 (18%)	52 (23%)	31 (17%)	39 (15%)	72 (18%)	62 (20%)	14 (14%)	
3rd quintile		15 (24%)	47 (21%)	36 (20%)	60 (22%)	96 (24%)	46 (15%)	20 (20%)	
4th quintile		10 (16%)	40 (18%)	46 (26%)	52 (19%)	74 (18%)	49 (16%)	18 (18%)	
5th quintile (Highest)		17 (27%)	28 (13%)	42 (23%)	67 (25%)	72 (18%)	54 (17%)	24 (24%)	

JPY, Japanese yen; SES, socioeconomic status.
Number of missing responses: i = 4; ii = 83.
P values were calculated with the chi-squared test.
